# Are young Iranian women with metabolically healthy obesity at increased risk of CVD incidence?

**DOI:** 10.1590/1677-5449.190106

**Published:** 2020-09-14

**Authors:** Seyed Ahmad Hosseini, Vahideh Aghamohammadi, Damoon Ashtary-Larky, Meysam Alipour, Matin Ghanavati, Nasrin Lamuchi-Deli

**Affiliations:** 1 Ahvaz Jundishapur University of Medical Sciences, Hyperlipidemia Research Center, Ahvaz, Iran.; 2 Ahvaz Jundishapur University of Medical Sciences, Nutrition and Metabolic Diseases Research Center, Ahvaz, Iran.; 3 Khalkhal University of Medical Sciences, Department of Nutrition, Khalkahl, Iran.; 4 Ahvaz Jundishapur University of Medical Sciences, School of Medicine, Department of Clinical Biochemistry, Ahvaz, Iran.

**Keywords:** metabolically healthy obese, atherogenic index of plasma, cardiovascular disease, obeso metabolicamente saudável, índice aterogênico do plasma, doença cardiovascular

## Abstract

**Background:**

The association between the Metabolically Healthy Obese (MHO) phenotype in the absence of metabolic syndrome and subsequent cardiovascular disease remains unclear.

**Objectives:**

We examined the association between MHO and CVD risk in young Iranian women.

**Methods:**

We studied 183 women aged 20-35 years from a population of 308 candidates. We classified participants into 4 phenotypes. We measured body composition, blood pressure, and biochemical factors in all participants.

**Results:**

The Metabolically Healthy Normal Weight (MHNW) and Normal Weight Obese (NWO) phenotypes had no statistical differences in any biochemistry variables. FBS, TG, LDL/HDL, Cholesterol/HDL, hs-CRP, and atherogenic index of plasma (AIP) were all higher in Metabolically Unhealthy Obese (MUO) than MHO individuals, whereas HDL was higher in MHO than in MUO individuals. LDL/HDL and hs-CRP were higher in MHO participants than MHNW participants, whereas HDL-c was higher in MHNW than MHO.

**Conclusions:**

Results of the present study demonstrate that young women displaying the MHO phenotype have a favorable metabolic profile as shown by lower FBS, TG, LDL-c/HDL, Cho/HDL, hs-CRP, and AIP and higher HDL levels than the MUO phenotype. However, MHO individuals were still at greater risk of CVD incidence (lower HDL and higher hs-CRP levels) than MHNW individuals.

## Resumo

**Contexto:**

A associação entre o fenótipo obeso metabolicamente saudável (OMS) na ausência de síndrome metabólica e doença cardiovascular subsequente permanece incerta.

**Objetivos:**

Examinamos a associação entre o fenótipo OMS e risco de DCV em jovens iranianas.

**Métodos:**

Analisamos 183 mulheres com idade de 20-35 anos de uma população de 308 candidatas. Classificamos as participantes em quatro fenótipos. Mensuramos composição corporal, pressão arterial e fatores bioquímicos em todas as participantes.

**Resultados:**

Os fenótipos com peso normal metabolicamente saudável (PNMS) e obeso com peso normal não apresentaram diferenças estatísticas em nenhuma das variáveis bioquímicas. Os níveis de glicemia sanguínea em jejum (GSJ), triglicerídeos (TG), relação LDL/HDL, HDL, proteína C reativa ultrassensível (PCR-us) e índice aterogênico do plasma (IAP) foram mais elevados em obesas metabolicamente não saudáveis (OMNS) do que em indivíduos OMSs, enquanto o HDL foi maior em OMSs do que em indivíduos OMNSs. A relação LDL/HDL e o nível de PCR-us foram mais elevados em participantes OMSs do que em participantes com PNMS, enquanto o HDL foi maior naquelas com PNMS do que nas OMSs.

**Conclusões:**

Os resultados do presente estudo demonstram que mulheres jovens com o fenótipo OMS têm um perfil metabólico favorável, conforme demonstrado pelos níveis menores de GSJ, TG, relação LDL/HDL, HDL, PCR-us e IAP e pelos níveis maiores de HDL em comparação às mulheres com o fenótipo OMNS. Entretanto, indivíduos OMSs ainda apresentavam maior risco de DCV incidente (níveis menores de HDL e maiores de PCR-us) do que indivíduos com PNMS.

## INTRODUCTION

Obesity is a key risk factor for cardiovascular diseases (CVD), diabetes, hypertension, various types of cancer, mental health, and increased mortality. The obesity rate has tripled over the last two decades in developing countries, including Iran.^1^ ^,^ ^2^

However, not all obese individuals show an increased risk of mortality. The metabolically healthy obesity (MHO) phenotype has been recognized since the 1980s and encompasses obese individuals who are metabolically healthy despite having an excessive store of body fat and elevated BMI.^3^ Individuals with MHO exhibit a favorable metabolic profile that is determined by a high level of insulin sensitivity, lack of hypertension, and favorable lipid, and inflammatory profiles.^3^ ^,^ ^4^ The MHO phenotype is the result of various underlying mechanisms and interactions between genetic, behavioral, and environmental agents that have not been elucidated.^5^ The results of previous studies evaluating the effect of MHO on health outcomes remain controversial.^6^ ^-^ ^8^ There is no consensus on unique criteria that can be used to define MHO and consequently prevalence rates of the MHO phenotype differ considerably among studies (6 to 40% in the obese population).^9^ The main obstacles to estimating the true prevalence of MHO are related to the criteria used to define it, the study design, and other factors such as ethnicity, sex, age, and lifestyle.^6^ ^-^ ^8^ Considering the number of serious health problems associated with obesity, research studying the MHO phenotype may help to identify at-risk obese individuals, support development of better interventions for obese patients, and lead to a novel comprehension of the pathophysiology of obesity.

Since the association between presence of MHO in the absence of metabolic syndrome and subsequent cardiovascular disease remains unclear, we examined the association between MHO and CVD risk in young Iranian women.

## METHODS

### Participants

The present cross-sectional study was carried out with subjects who were referred to a nutrition clinic, in Ahvaz, Iran. We studied 183 women aged 20-35 years who were selected from a population of 308 candidates. We excluded 125 women who met exclusion criteria including pregnancy, breastfeeding, consumption of any drugs, eating disorders, diabetes, cardiovascular disease, kidney disorders, thyroid disorders, digestive and respiratory diseases, cancer, consumption of more than 300 mg of caffeine daily, and moderate or severe physical activity). Inclusion criteria were regular 28-d menstrual cycles, no physical activity, no smoking, no alcohol consumption, no use of any supplements, and no weight changes in the preceding 6 months. All 183 women signed written informed consent forms. The study protocol was approved by the Ethics Committee at the Ahvaz Jundishapur University of Medical Sciences (IR.AJUMS.REC.1394.489). Participants with metabolic syndrome were diagnosed according to the NCEP ATP III definition, i.e., meeting at least three of the following criteria: waist circumference ≥ 35 inches (women), blood pressure ≥ 130/85 mmHg, fasting triglyceride (TG) level ≥ 150 mg/dL, fasting high-density lipoprotein (HDL) cholesterol level less than 50 mg/dL (for women), and fasting blood sugar (FBS) ≥ 100 mg/dL.^10^ We classified participants according to 4 phenotypes based on body mass index (BMI) and metabolic syndrome criteria (Table 1).

**Table 1:** Criteria used for diagnosis of metabolically obese phenotypes.

**Inflammatory obese phenotypes**	**Obesity status**	**Metabolic syndrome**
Metabolically healthy normal weight (MHNW)	< 25 (kg/m^2^)	No
Normal weight obese (NWO)	< 25 (kg/m^2^) and body fat ˃ 30%	Yes/No
Metabolically healthy obese (MHO)	≥ 25 (kg/m^2^)	No
Metabolically unhealthy obese (MUO)	≥ 25 (kg/m^2^)	Yes

### Measurements

A direct segmental multi-frequency bioelectrical impedance method (Inbody 270, Biospace, Korea) was used to calculate body weight and body composition. Waist circumference (WC) was determined in the standing position using a tape with an accuracy of 1.0 cm, above the iliac crest and just below margin of the lowest rib, at the end of the normal expiration. For the hip circumference measurement, the tape was put around the point with the maximum circumference over the buttocks.^11^ The waist-to-hip ratio (WHR) was calculated as waist measurement divided by hip measurement (W/H). Blood pressure was determined using an automatic blood pressure monitor (BM65, Beurer, Germany) after subjects had been at rest for more than 10 minutes. Measurements were taken in triplicate and the mean was considered for each subject. In this study, RMR was measured by indirect calorimetry (FitMate, Cosmed, Rome, Italy), using resting oxygen uptake (VO2).

For biochemical measurements, 8 cc fasting blood samples were drawn from each subject between days 8 and 12 of their preovulation phase. Blood glucose, total cholesterol, LDL-c, HDL-c, and triglycerides were quantified by enzymatic methods with kits from Pars- Azmoon (Tehran, Iran). Insulin concentration and high-sensitivity C-reactive protein (hsCRP) were measured with ELISA kits (Diaplus Inc., Canada). Homeostasis model assessment – insulin resistance (HOMA-IR) was determined using the following formula: 

$$fasting\;glucose\;\left(mg/dL\right)\;\times \;fasting\;insulin\;\left(\mu u/mL\right)/405$$

QUICKI was measured with the following formula = 

$$1\;/\;\left[log\;\left(fasting\;insulin\;\left(\mu U\;mL\right)\right)\;+\;log\;\left(fasting\;glucose\;\left(mg/\;dL\right)\right)\right]\;20\;/\;\left(fasting\;C-peptide\times \;fasting\;plasma\;glucose\right)$$

^12^

### Statistical analyses

All statistical analyses were carried out using IBM SPSS Statistics software version 24 (IBM SPSS Statistics, Armonk, USA). The normality of variables was established using the Kolmogorov- Smirnov test. ANOVA was applied to test differences within groups. Comparisons of variables that were different between groups were performed using the Tukey test for post-hoc analyses. A p value less than 0.05 was considered to be statistically significant.

## RESULTS

Table 2 lists anthropometric characteristics of the participants included in the analysis. A total of 44.8% of the participants had normal weight, and 55.2% were overweight or obese. There were no significant differences in age, height, diastolic blood pressure, or heart rate between groups. Weight, BMI, WC, HC, WHR, and RMR were highest in MUO, followed by MHO, NWO, and MHNW in that order. Total body water was significantly higher in obese groups (MUO, MHO) than in normal weight individuals. Systolic blood pressure was significantly higher in MUO than in the other three groups (Table 2). As shown in Tables 3 and Table 4, the amount of fat in all parts of the body was higher in NWO than in MHNW, but this difference was not observed for LBMs. Body fat and LBM of all body parts was higher in obese groups (MUO and MHO) than in normal weight groups (NWO and MHNW). Also, body fat and LBM (expect fat free mass, left and right foot) were higher in MUO than MHO (Table 3 and Table 4). As shown in Tables 5 and Table 6, there were no significant differences between NHNW and NWO in lipid profile, glycemic status, or liver enzymes. The MUO group had significantly higher values for TG, VLDL, LDL/HDL, Cholesterol/HDL, AIP, FBS, and hsCRP than the other three groups. Furthermore, the lowest level of HDL was observed in the MUO group. LDL/HDL and hs-CRP were higher in MHO participants than in MHNW participants, whereas HDL-c was higher in MHNW than MHO.

**Table 2:** Anthropometric status by group.

**Value**	MHNW	NWO	MHO	MUO	P*
	(n=53)	(n=29)	(n=57)	(n=44)	
Age	26.36 ± 5.01	27.21 ± 4.61	28.44 ± 4.53	27.09 ± 4.30	0.13
Weight	56.33 ± 6.70^b^ ^,c^	58.69 ± 4.58^d^ ^,e^	83.93 ± 14.85^f^	93.12 ± 14.91	< 0.001
Height	159.44 ± 5.42	158.62 ± 4.93	159.62 ± 5.31	158.30 ± 4.83	0.55
LBM	21.02 ± 2.29^b,^ c	20.42 ± 2.05^d,^ e	25.80 ± 4.74	26.75 ± 3.50	< 0.001
FM	17.39 ± 4.23^b,c^	20.72 ± 2.13^d,e^	37.77 ± 10.35^f^	45.14 ± 10.13	< 0.001
TBW	28.52 ± 2.80^b,c^	27.77 ± 2.48^d,e^	33.85 ± 4.51	35.21 ± 4.26	< 0.001
FFM	38.90 ± 3.81^b,c^	37.91 ± 3.39^d,e^	45.62 ± 5.37	47.92 ± 5.77	< 0.001
BMI	22.14 ± 1.85^b,c^	23.29 ± 1.21^d,e^	33.19 ± 6.37	37.17 ± 5.53	< 0.001
BFP	30.60 ± 5.66^a^ ^,b,c^	35.32 ± 2.45^d,e^	44.63 ± 5.00	48.00 ± 4.21	< 0.001
WHR	0.76 ± 0.06^b,c^	0.77 ± 0.05^d,e^	0.86 ± 0.07	0.87 ± 0.05	< 0.001
WC	71.20 ± 5.65^b,c^	74.25 ± 4.53^d,e^	95.82 ± 12.90^f^	102.29 ± 9.79	< 0.001
HC	93.04 ± 5.09^b,c^	95.72 ± 4.48^d,e^	111.03 ± 9.11^f^	116.25 ± 7.40	< 0.001
HR	93.96 ± 14.07	90.34 ± 13.42	92.75 ± 12.75	88.41 ± 11.23	0.16
SBP	113.77 ± 11.09^c^	115.70 ± 11.27^e^	115.37 ± 14.31^f^	129.28 ± 14.18	< 0.001
DBP	75.94 ± 9.96	76.93 ± 10.81	75.03 ± 15.18^f^	81.83 ± 13.74	0.05
RMR	1333.01 ± 81.34^b,c^	1310.25 ± 81.34^d, e^	1501.83 ± 126.47	1547.36 ± 136.94	< 0.001

MHNW = metabolically healthy normal weight; NWO = normal weight obese; MHO = metabolically healthy obese; MUO = metabolically unhealthy obese; LBM = lean body mass; FM = fat mass; TBW = total body water; FFM = fat free mass; BMI = body mass index; BFP = percentage of body fat; WHR = waist-to-hip ratio; WC = waist circumference; HC = hip circumference; HR = heart rate; SBP = systolic blood pressure; DBP = diastolic blood pressure; RMR = resting metabolic rate.

* P-value of the comparison between groups.

a significant difference between MHNW and NWO;

b significant difference between MHNW and MHO;

c significant difference between MHNW and MUO;

d significant difference between NWO and MHO;

e significant difference between NWO and MUO;

f significant difference between MHO and MUO.

**Table 3:** Body composition by group.

**Value**	MHNW	NWO	MHO	MUO	P*
	(n=53)	(n=29)	(n=57)	(n=44)	
LBM (left arm)	1.82 ± 0.27^a^ ^,b^	1.78 ± 0.25^c^^,^ d	2.46 ± 0.42^e^	2.66 ± 0.43	< 0.001
LBM (right arm)	1.85 ± 0.28^a,^ b	1.81 ± 0.27^c,d^	2.47 ± 0.41^e^	2.68 ± 0.42	< 0.001
LBM (trunk)	17.33 ± 1.80^a,b^	17.15 ± 1.74^c,d^	21.14 ± 2.50^e^	22.37 ± 2.61	< 0.001
LBM (left foot)	5.89 ± 0.75^a,b^	5.71 ± 0.66^c,d^	6.83 ± 0.86	7.18 ± 0.99	< 0.001
LBM (right foot)	5.89 ± 0.75^a,b^	5.72 ± 0.67^c,d^	6.89 ± 0.85	7.20 ± 1.04	< 0.001
LBM (kg)	21.02 ± 2.29^a,b^	20.42 ± 2.05^c,d^	25.80 ± 4.74	26.75 ± 3.50	< 0.001
FFM	38.90 ± 3.81^a,b^	37.91 ± 3.39^c,d^	45.62 ± 5.37	47.92 ± 5.77	< 0.001

MHNW = metabolically healthy normal weight; NWO = normal weight obese; MHO = metabolically healthy obese; MUO = metabolically unhealthy obese; LBM = lean body mass; FFM = fat free mass; BMI = body mass index; BFP = percentage of body fat.

* P-value of the comparison between groups.

a significant difference between MHNW and MHO;

b significant difference between MHNW and MUO;

c significant difference between NWO and MHO;

d significant difference between NWO and MUO;

e significant difference between MHO and MUO.

**Table 4:** Body fat status by group.

**Value**	MHNW	NWO	MHO	MUO	P*
	(n=53)	(n=29)	(n=57)	(n=44)	
FM (left arm)	1.20 ± 0.36^a^ ^,b,c^	1.48 ± 0.21^d^ ^,e^	3.55 ± 1.58^f^	4.55 ± 1.58	< 0.001
BFP (left arm)	37.83 ± 7.96^a,^ b ^,c^	43.99 ± 4.64^d,^ e	55.62 ± 7.50^f^	60.32 ± 7.07	< 0.001
FM (right arm)	1.17 ± 0.35^a,b,^ c	1.46 ± 0.19^d,e^	3.51 ± 1.56^f^	4.52 ± 1.60	< 0.001
BFP (right arm)	36.94 ± 8.01^a,b,c^	43.25 ± 4.62^d,e^	55.22 ± 7.77^f^	61.60 ± 3.54	< 0.001
FM (trunk)	8.56 ± 2.35^a,b,c^	10.41 ± 1.23^d,e^	18.53 ± 4.31^f^	21.31 ± 3.80	< 0.001
BFP (trunk)	31.40 ± 5.99^a,b,c^	36.38 ± 2.41^d,e^	44.75 ± 3.87^f^	47.12 ± 3.08	< 0.001
FM (left foot)	2.69 ± 0.59^a,b,c^	3.15 ± 0.32^d,e^	5.49 ± 1.51^f^	6.57 ± 1.71	< 0.001
BFP (left foot)	30.02 ± 5.32^a,b,c^	34.26 ± 2.88^d,e^	42.52 ± 5.07^f^	45.80 ± 4.88	< 0.001
FM (right foot)	2.70 ± 0.58^a,b,c^	3.15 ± 0.31^d,e^	5.53 ± 1.53^f^	6.62 ± 1.75	< 0.001
BFP (right foot)	30.11 ± 5.01^a,b,c^	34.27 ± 2.83^d,e^	42.45 ± 5.15^f^	45.88 ± 4.82	< 0.001
FM/LBM	0.83 ± 0.22^a,b,c^	1.02 ± 0.11^d,e^	1.46 ± 0.31^f^	1.69 ± 0.27	< 0.001
FM/LBM (left arm)	0.67 ± 0.23^a,b,c^	0.84 ± 0.15^d,e^	1.40 ± 0.44^f^	1.69 ± 0.47	< 0.001
FM/LBM (right arm)	0.65 ± 0.23^a,b,c^	0.82 ± 0.15^d,e^	1.38 ± 0.45^f^	1.67 ± 0.48	< 0.001
FM/LBM (trunk)	0.50 ± 0.13^a,b,c^	0.60 ± 0.06^d,e^	0.86 ± 0.13^f^	0.95 ± 0.11	< 0.001
FM/LBM (left foot)	0.46 ± 0.12^a,b,c^	0.55 ± 0.07^d,e^	0.80 ± 0.17^f^	0.91 ± 0.17	< 0.001
FM/LBM (right foot)	0.46 ± 0.11^a,b,c^	0.55 ± 0.07^d,e^	0.79 ± 0.17^f^	0.91 ± 0.17	< 0.001
FM (kg)	17.39 ± 4.23^b,c^	20.72 ± 2.13^d,e^	37.77 ± 10.35^f^	45.14 ± 10.13	< 0.001
BF (%)	30.60 ± 5.66^a,b,c^	35.32 ± 2.45^d,e^	44.63 ± 5.00	48.00 ± 4.21	< 0.001

MHNW = metabolically healthy normal weight; NWO = normal weight obese; MHO = metabolically healthy obese; MUO = metabolically unhealthy obese; LBM = lean body mass; FM = fat mass; BFP = percentage of body fat; BF = body fat.

* P-value of the comparison between groups.

a significant difference between MHNW and NWO;

b significant difference between MHNW and MHO;

c significant difference between MHNW and MUO;

d significant difference between NWO and MHO;

e significant difference between NWO and MUO;

f significant difference between MHO and MUO.

**Table 5:** Lipid profiles by group.

**Value**	MHNW	NWO	MHO	MUO	**P** *
	(n=53)	(n=29)	(n=57)	(n=44)	
Cholesterol	174.13 ± 24.79	179.89 ± 29.15	180.86 ± 30.31	187.45 ± 36.18	0.19
Triglycerides	100.52 ± 49.93^b^	102.79 ± 64.52^c^	100.94 ± 33.06^d^	157.50 ± 47.96	< 0.001
HDL	52.18 ± 7.56^a^ ^,b^	49.10 ± 7.59^c^	47.77 ± 9.13^d^	37.79 ± 6.04	< 0.001
LDL	101.15 ± 23.66^b^	107.20 ± 28.71	113.80 ± 28.28	120.81 ± 34.83	0.008
LDL/HDL	2.01 ± 0.77^a,b^	2.28 ± 0.94^c^	2.55 ± 0.97^d^	3.29 ± 1.10	< 0.001
Cholesterol/HDL	3.45 ± 1.04^b^	3.82 ± 1.30^c^	3.99 ± 1.08^d^	5.10 ± 1.27	< 0.001
VLDL	20.10 ± 9.98^b^	20.55 ± 12.90^c^	20.19 ± 6.61^d^	31.50 ± 9.59	< 0.001
AIP	0.61 ± 0.19^b^	0.63 ± 0.24^c^	0.67 ± 0.17^d^	0.96 ± 0.17	< 0.001

MHNW = metabolically healthy normal weight; NWO = normal weight obese; MHO = metabolically healthy obese; MUO = metabolically unhealthy obese.; HDL = High-density lipoprotein; LDL = Low-density lipoprotein; VLDL = Very-low-density lipoprotein; AIP = Atherogenic index of plasma.

* P-value of the comparison between groups.

a significant difference between MHNW and MHO;

b significant difference between MHNW and MUO;

c significant difference between NWO and MUO;

d significant difference between MHO and MUO.

**Table 6:** Biochemical factors by group.

**Value**	MHNW	NWO	MHO	MUO	P*
	(n=53)	(n=29)	(n=57)	(n=44)	
AST	24.26 ± 18.46	26.55 ± 24.10	23.63 ± 9.22	23.81 ± 12.29	0.86
ALT	20.03 ± 11.06	21.06 ± 13.94	23.94 ± 9.93	23.95 ± 13.67	0.25
ALP	181.01 ± 42.82	185.58 ± 37.39	184.51 ± 59.87	195.38 ± 64.40	0.6
FBS	87.28 ± 9.27^b^	89.27 ± 9.23	88.31 ± 9.28^d^	93.68 ± 11.14	0.01
Insulin	9.70 ± 5.07	10.69 ± 5.96	11.32 ± 6.52	12.61 ± 7.96	0.17
HOMA_IR	1.23 ± 0.63	1.36 ± 0.74	1.43 ± 0.80	1.62 ± 1.01	0.13
HOMA-s%	99.58 ± 45.20	95.85 ± 53.09	90.73 ± 48.47	82.03 ± 41.41	0.31
HOMA-b%	119.23 ± 48.13	121.72 ± 56.15	129.34 ± 58.50	122.13 ± 49.60	0.78
hsCRP	1.56 ± 1.24^a^^,^ b	2.08 ± 1.10^c^	4.09 ± 5.18^d^	6.64 ± 5.54	< 0.001

MHNW = metabolically healthy normal weight; NWO = normal weight obese; MHO = metabolically healthy obese; MUO = metabolically unhealthy obese; AST = aspartate aminotransferase; ALT = alanine aminotransferase; ALP = alkaline phosphatase; FBS = fasting blood sugar; HOMA_IR = Homeostatic Model Assessment for Insulin Resistance; HOMA-s% = HOMA of insulin sensitivity; HOMA-b% = HOMA of β-cell function; hsCRP = High sensitivity C-reactive protein.

* P-value of the comparison between groups.

a significant difference between MHNW and MHO;

b significant difference between MHNW and MUO;

c significant difference between NWO and MUO;

d significant difference between MHO and MUO.

## DISCUSSION

Our analysis revealed that MHO individuals were at increased risk of CVD incidents, compared with normal weight individuals without metabolic syndrome. However, MHO individuals display a partially more favorable metabolic profile than MUO individuals. Despite the increasing number of studies evaluating MHO, investigations of the association between metabolically healthy obesity and CVD risk have reported controversial results. Several studies have observed no elevated risk of CVD in MHO individuals, whereas some other studies have shown an elevated risk of CVD in this phenotype.^13^ ^-^ ^15^ In one study of women aged 45 years and over, with 10 years of follow-up, no increased CVD risk for MHO phenotypes was observed.^13^ In another study, in adults aged 35-55 years, it was shown that MHO individuals were at increased risk of CVD incidents, compared with MHNW individuals.^14^ Karelis et al.^15^ indicated that postmenopausal women displaying the MHO phenotype also have a favorable inflammation profile, as shown by lower CRP and α-1 antitrypsin levels compared with insulin-resistant women. These authors suggested that a satisfactory inflammation profile, as verified by low CRP levels, could play a role in the protective profile of the MHO individual and that this may be related metabolically to a lower risk of cardiovascular disease.^15^ Also, we found that a low inflammation state was a part of the protective profile of the MHO subgroup, when compared to the MUO subgroup. We observed a lower hs-CRP concentration in MHO participants than MUO participants (4.09 ± 5.18 vs 6.64 ± 5.54, respectively). However, the MHO subgroup’s mean hs-CRP level was higher than the JUPITER threshold, ≥2mg/L and also higher than that of the MHNW subgroup.^16^ The second favorable aspect of the MHO metabolic profile compared to the MUO profile was a higher HDL level, and lower levels of fasting TG, LDL/HDL, and Cholesterol/HDL. An adequate lipid profile has been reported as a protective factor for cardiovascular disease.^17^ Another variable associated with a more favorable metabolic profile in MHO in compared to MUO was FBS. We also found that young MHO women had no increased risk of developing type 2 diabetes compared with MHNW. Finally, one other protective variable was lower AIP in MHO participants compared with MUO subjects. This variable was not statistically different between MHO and MHNW or NWO participants. In a study by Koborová et al.,^18^ AIP was significantly lower in metabolically healthy obese women than in unhealthy centrally obese women, which is similar to our findings. Also, a study by Chhezom showed that obese individuals had a significantly higher atherogenic index of plasma compared to normal weight and overweight individuals.^19^ Recently, AIP was identified as a useful and novel marker for the risk of atherosclerosis and cardiovascular disease.^20^ This marker reflects the delicate metabolic interactions within the whole lipoprotein complex and is a better predictor of atherogenic risk than triglyceride and HDL-C levels evaluated separately.^21^

It is likely that MHO is a transient condition.^5^ ^,^ ^22^ Thus, in MHO individuals, sustaining factors such as a healthy diet, smoking cessation, preservation of metabolic health, and physical activity may prevent progression to the MUO phenotype.^4^ ^-^ ^7^ Dhana et al.^23^ suggested that the metabolic status of MHO individuals should be reassessed on a regular basis.

We employed a cross-sectional approach, so we couldn’t establish relationships of causality. Despite this limitation, our results are strengthened by measurement of body composition, liver function enzymes, complete lipid profile, glycemic indexes and inflammatory markers in young Iranian women.

In conclusion, results of the present study demonstrate that young women displaying the MHO phenotype have a favorable metabolic profile as shown by lower FBS, TG, LDL-c/HDL-c, Cho/HDL-c, hs-CRP, and AIP, and higher HDL-c levels than those with the MUO phenotype. However, MHO individuals were nevertheless at increased risk of CVD incident (lower HDL-C and more hs-CRP level), compared with MHNW individuals. As mentioned above, the inconsistent definition of MHO among studies is the main obstacle to advancing our understanding of the MHO phenotype. It is therefore necessary to standardize the definition of MHO.
